# Functional characterization of a three-component regulatory system involved in quorum sensing-based regulation of peptide antibiotic production in *Carnobacterium maltaromaticum*

**DOI:** 10.1186/1471-2180-6-93

**Published:** 2006-10-20

**Authors:** Bettina H Rohde, Luis EN Quadri

**Affiliations:** 1 Department of Microbiology and Immunology, Weill Medical College of Cornell University, New York City, USA; 2 GATC Biotech AG, Konstanz, Germany; 3 Molecular Biology Program and Tri-Institutional Training Program in Chemical Biology, Weill Graduate School of Medical Sciences of Cornell University, New York City, USA

## Abstract

**Background:**

Quorum sensing is a form of cell-to-cell communication that allows bacteria to control a wide range of physiological processes in a population density-dependent manner. Production of peptide antibiotics is one of the processes regulated by quorum sensing in several species of Gram-positive bacteria, including strains of *Carnobacterium maltaromaticum*. This bacterium and its peptide antibiotics are of interest due to their potential applications in food preservation. The molecular bases of the quorum sensing phenomenon controlling peptide antibiotic production in *C. maltaromaticum *remain poorly understood. The present study was aimed at gaining a deeper insight into the molecular mechanism involved in quorum sensing-mediated regulation of peptide antibiotic (bacteriocin) production by *C. maltaromaticum*. We report the functional analyses of the CS (autoinducer)-CbnK (histidine protein kinase)-CbnR (response regulator) three-component regulatory system and the three regulated promoters involved in peptide antibiotic production in *C. maltaromaticum *LV17B.

**Results:**

CS-CbnK-CbnR system-dependent activation of carnobacterial promoters was demonstrated in both homologous and heterologous hosts using a two-plasmid system with a β-glucuronidase (GusA) reporter read-out. The results of our analyses support a model in which the CbnK-CbnR two-component signal transduction system is necessary and sufficient to transduce the signal of the peptide autoinducer CS into the activation of the promoters that drive the expression of the genes required for production of the carnobacterial peptide antibiotics and the immunity proteins that protect the producer bacterium.

**Conclusions:**

The CS-CbnK-CbnR triad forms a three-component regulatory system by which production of peptide antibiotics by *C. maltaromaticum *LV17B is controlled in a population density-dependent (or cell proximity-dependent) manner. This regulatory mechanism would permit the bacterial population to synchronize the production of peptide antibiotics and immunity proteins. Such a population-wide action would afford a substantial peptide antibiotic production burst that could increase the ability of the bacterium to inhibit susceptible bacterial competitors. Finally, our CS-CbnK-CbnR-based two-plasmid expression system represents a suitable genetic tool for undertaking structure-function relationship analyses to map the amino acid residues in the components of the CS-CbnK-CbnR system that are required for biological activity. This plasmid system also has potential as a starting point for developing alternative vectors for controlled gene expression in *C. maltaromaticum, Lactococcus lactis*, and related lactic acid bacteria.

## Background

Numerous bacterial processes are controlled in a population density-dependent manner. This phenomenon is based on a form of cell-to-cell communication that is referred to as quorum sensing, and it regulates microbial processes of medical, economic, and ecological relevance. Among these processes are development of genetic competence, sporulation, formation of biofilm, bioluminescence, expression of virulence factors, and production of antibiotics [1-6]. Deciphering the molecular bases of quorum sensing is important for understanding and manipulating the behavior of microbes in their natural and artificial niches. The practical implications of gaining a better understanding of quorum sensing and other types of bacterial cell-to-cell communication mechanisms are foreseen in areas as diverse as identification of new targets amenable to therapeutic intervention to treat human diseases, production of foods, feeds, and pharmacologically relevant compounds, and biological control of crop and animal pathogens [7-9].

Quorum sensing allows unicellular organisms to modulate a wide range of physiological responses in a population density-dependent manner, thereby enabling unicellular organisms to evoke cell population-wide synchronous responses that are typical of multicellular organisms. The phenomenon of quorum sensing is mediated by secreted small signal molecules that are usually referred to as autoinducers [1-6]. From the chemical nature point of view, the majority of the known autoinducers belong to one of two types: *N-*acyl homoserine lactones, which are most commonly utilized as communication signals by Gram-negative bacteria; or (oligo/poly)peptides, which are characteristically found in cell-to-cell communication systems of Gram-positive bacteria [1-6]. Other autoinducer types have also been identified, including 2-heptyl-3-hydroxy-quinolone and 4,5-dihydroxy-2,3-petanedione (which spontaneously cyclizes into furanones in equilibrium) [1,3]. Following secretion, autoinducers accumulate in the environment and, when a threshold concentration is reached, trigger signal transduction events in the target cells that lead to changes in the expression levels of genes that are under autoinducer-dependent control. This cellular response requires interaction of the autoinducer with a specific protein receptor. The receptors of *N*-acyl homoserine lactone autoinducers are typically cytoplasmic proteins that become competent transcriptional activators upon binding of the cognate autoinducer ligand. In the case of peptide autoinducers, the receptors are typically membrane-bound histidine protein kinases of two-component signal transduction systems that transduce signals via a phosphorylation cascade and response regulator proteins that exert transcriptional control on the target genes.

The production of peptide antibiotics (bacteriocins) is a process that is regulated by peptide autoinducer-dependent quorum sensing in several species of Gram-positive bacteria [6,10,11]. These peptide antibiotics are diverse in terms of structure, mode of action, antimicrobial potency, spectrum of antimicrobial activity, and industrial applicability [6,11]. Among these peptide antibiotic-producing Gram-positive bacteria are species of the genus *Carnobacterium*. Members of this genus were originally included in the genus *Lactobacillus *[12], and they can be found in a wide range of natural niches, and as adventitious microflora in a variety of food products [13]. *C. maltaromaticum *LV17B, a bacterium isolated from refrigerated fresh meat [14], produces the peptide antibiotics known as carnobacteriocin B2 (48 amino acid residues) and carnobacteriocin BM1 (43 amino acid residues) [15]. The carnobacteriocins display potent antimicrobial activity against several Gram-positive bacteria, including, most significantly, the nosocomial pathogen *Enterococcus faecalis *and the food-borne pathogen *Listeria monocytogenes *[15]. Like other peptide antibiotics and their producing lactic acid bacteria, the carnobacteriocins and their producer, *C. maltaromaticum*, are candidate food biopreservative agents for exploration of preservation strategies that suppress or reduce the growth of bacterial pathogens and bacteria with high spoilage potential [16].

**Figure 1:**
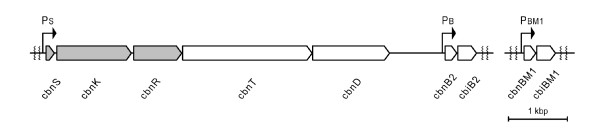
**Gene loci involved in carnobacteriocin B2 and BM1 production and immunity**. The locus in the plasmid pCP40 (left) contains the *cbnS-cbnK-cbnR *three-component regulatory system gene cassette (genes colored in gray), *cbnT *and *cbnD*, encoding a peptide autoinducer and peptide antibiotic secretion/processing system, *cbnB2*, encoding the precursor of carnobacteriocin B2, and *cbiB2*, encoding carnobacteriocin B2 immunity protein. The chromosomal locus (right) includes *cbnBM1*, encoding the precursor of carnobacteriocin BM1, and *cbiBM1*, encoding carnobacteriocin BM1 immunity protein. The positions of the promoters PS, PB, and PBM1 are shown.

In *C. maltaromaticum *LV17B, the genes required for carnobacteriocin production are distributed in two loci, one in a 61-kb plasmid (pCP40) and another in the chromosome [15,17-19] (Figure 1). The locus in pCP40 includes the *cbnS-cbnK-cbnR *gene triad, which is predicted to encode a so-called three-component regulatory system. Three-component regulatory systems composed of peptide autoinducer-sensor kinase-response regulator triads are emerging as important regulatory systems in Gram-positive bacteria with low GC content [20]. The gene *cbnS *encodes the precursor of the peptide autoinducer named CS (24 amino acid residues). This autoinducer precursor has a leader peptide of 17-amino acid residues that is cleaved off concomitantly with autoinducer secretion. The genes *cbnK *and *cbnR *encode a histidine protein kinase of the subfamily 10 and a response regulator protein, respectively, of a two-component signal transduction system [21,22]. The CbnK-CbnR system is the proposed peptide autoinducer sensing and signal transducing apparatus controlling carnobacteriocin production.

In this study, we characterized the regulatory circuit that is involved in CS-dependent regulation of peptide antibiotic production in *C. maltaromaticum *LV17B. We report the dissection and functional reconstitution of the CS-CbnK-CbnR three-component regulatory system in the homologous *C. maltaromaticum *host and in the heterologous *Lactococcus lactis *host. We also present the functional analysis of three *C. maltaromaticum *promoters that are regulated by the CS-CbnK-CbnR system.

## Results and discussion

### Dissection and functional reconstruction of the CS-CbnK-CbnR three-component regulatory system in *Carnobacterium maltaromaticum *LV17C

The carnobacteriocins produced by the lactic acid bacterium *C. maltaromaticum *LV17B have potent antimicrobial activity against several Gram-positive bacteria, including important human pathogens and bacteria with high food-spoilage properties [15]. *C. maltaromaticum *LV17B and its carnobacteriocins are candidate biopreservative agents for exploration of novel food preservation strategies. With this consideration in mind, it is important to decipher the molecular mechanism controlling carnobacteriocin production. To this end, we sought to validate the functioning of the hypothesized CS-CbnK-CbnR three-component regulatory system encoded in the carnobacteriocin production locus in the plasmid pCP40 of *C. maltaromaticum *LV17B.

To functionally characterize the CS-CbnK-CbnR system, we first constructed two series of plasmids suitable for synchronous replication in *C. maltaromaticum *that permitted us to investigate the activity of the regulatory system by utilizing a GusA reporter read-out (Table 1). Members of one of these plasmid series were derived from the expression vector pIL253 and constitutively expressed a wild-type *cbnK-cbnR *gene cassette (Figure 1), or a gene cassette with a mutation in one of the regulatory genes. Three pIL253 derivatives expressing regulatory proteins were constructed: pBRKR (expressing CbnK and CbnR wild-type); pBRKA239R (expressing CbnK His 239 to Ala mutant and CbnR); and pBRKRN62 (expressing CbnK and CbnR Asp 62 to Asn mutant). The amino acid substitution in each regulatory protein was made at its predicted phosphorylation site, which was previously identified by comparative sequence analysis with known histidine protein kinases or response regulator proteins [18]. Members of the other plasmid series were derived from the promoter screening vector pNZ273, and contained the promoter-less reporter gene *gusA *of pNZ273 fused downstream of the suspected CbnKR-regulated promoters PBM1 (pLQPBM1 plasmid), PB (pLQPB plasmid), or PS (pLQPS plasmid) (Figure 1). Each of the pIL253 derivatives or pIL253 was introduced in *C. maltaromaticum *LV17C *(C. maltaromaticum *LV17B without the plasmid pCP40). Prior to introduction of each of these plasmids in *C. maltaromaticum *LV17C, the strain was transformed with the pNZ273 derivatives or pNZ273, thus creating a set of two plasmid-containing *C. maltaromaticum *LV17C transformants suitable for the functional investigation of the CS-CbnK-CbnR three-component regulatory system.

**Figure 2:**
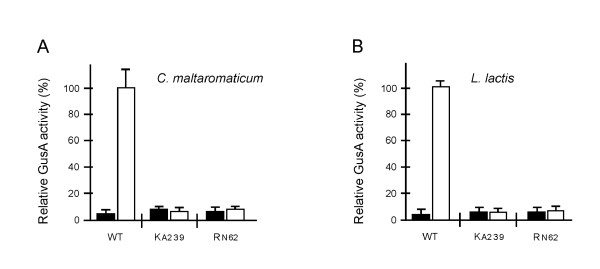
**Functional analysis of the CS-CbnK-CbnR three-component regulatory system in a homologous (A) and heterologous (B) host**. *C. maltaromaticum *LV17C and *L. lactis *MG1363 transformants carried pLQPBM1 and either pBRKR (WT columns), pBRKA239R (KA239 columns), or pBRKRN62 (RN62 columns). The transformants were treated with 20 μg/ml of CS (white columns) or 0.05% trifluoroacetic acid (black columns). GusA activity was determined as described in Methods. Means ± standard error of triplicate assays are shown.

We first investigated whether the pBRKR/pLQPBM1* C. maltaromaticum *LV17C transformant, carrying the wild-type *cbnK-cbnR *gene cassette, had a functional CS-triggered signal transduction cascade capable of mediating the activation of the PBM1 promoter. To this end, we examined the effect of CS treatment on the GusA activity of this transformant. The comparison between the GusA activity in cell suspensions of CS-treated cultures and the GusA activity in cell suspensions of 0.05% trifluoroacetic acid-treated cultures revealed that the pBRKR/pLQPBM1 transformant had a robust induction of reporter activity only when treated with the peptide autoinducer (Figure 2A). In contrast, irrespective of treatment, no significant GusA activity was detected in the pIL253/pLQPBM1* C. maltaromaticum *LV17C transformant or the pBRKR/pNZ273 *C. maltaromaticum *LV17C transformant (not shown). These results clearly demonstrated that the pBRKR/pLQPBM1 transformant has a functional CbnK- and/or CbnR-dependent signal transduction system that is capable of activating transcription of the promoter PBM1 in response to CS.

Based on the sequence similarity of CbnK and CbnR to histidine protein kinases and response regulator proteins, respectively, of two-component regulatory systems, we speculated that both carnobacterial proteins would be required for CS-dependent induction of GusA activity, and that the CbnK-CbnR system would utilize a phosphorylation cascade to transduce the CS signal into promoter activation. To probe this hypothesis, we determined whether a site-specific mutation that removed the predicted phosphorylation site of CbnK (His 239) or of CbnR (Asp 62) would affect the ability of the CbnK-CbnR system to activate the promoter PBM1 in a CS-dependent manner. The analysis of the pBRKA239R/pLQPBM1* C. maltaromaticum *LV17C transformant showed that the substitution of His 239 of CbnK with Ala rendered a transformant with no GusA activity, irrespective of CS treatment. Likewise, examination of the pBRKRN62/pLQPBM1 *C. maltaromaticum *LV17C transformant indicated that the replacement of Asp 62 of CbnR with Asn abrogated the function of the CbnK-CbnR system, as judged by the lack of CS-induced GusA activity (Figure 2A). These results demonstrate that both CbnK and CbnR are required for CS-dependent activation of the promoter PBM1 in the *C. maltaromaticum *genetic background. Moreover, the fact that His 239 and Asp 62 are needed for biological activity of CbnK and CbnR, respectively, is in agreement with the sequence-based predicted role of each of these residues as a site for phosphorylation [18].

### Functional reconstruction of the CS-CbnK-CbnR three-component regulatory system in a heterologous host

To investigate the portability of the CS-CbnK-CbnR three-component regulatory system to a different genetic background, we assessed whether this regulatory system could be functionally reconstructed in a heterologous host. *L. lactis *was selected as a sensible host for these experiments since it is a lactic acid bacterium with a codon usage and a G + C content that is similar to that of *C. maltaromaticum*. More importantly, the pIL253 and pNZ273 vectors had been demonstrated to be suitable for utilization in *L. lactis *[23,24].

Each of the pIL253 derivatives expressing the carnobacterial regulatory proteins (pBRKR, pBRKA239R, and pBRKRN62) or pIL253 was introduced in *L. lactis *strains that were already transformed with pLQPBM1 (carrying the PBM1*-gusA *fusion) or pNZ273. This allowed us to generate a set of two plasmid-containing *L. lactis *transformants equivalent to the two plasmid-containing *C. maltaromaticum *transformants described above. The functionality of the CS-CbnK-CbnR system in the *L. lactis *transformants was examined with the same methodological approach utilized for the analysis of their *C. maltaromaticum *transformant counterparts. The analysis showed that the CS-triggered signal transduction cascade that leads to the activation of the PBM1 promoter was functional in *L. lactis*. Moreover, as seen in *C. maltaromaticum*, CS-triggered promoter activation required both CbnK and CbnR (Figure 2B). Overall, these results clearly demonstrate that the CbnK-CbnR two-component signal transduction system is necessary and sufficient to transduce the signal of the peptide autoinducer CS into activation of the promoter PBM1 (and likely the promoters PS and PB) in both the homologous *C. maltaromaticum *host and the heterologous *L. lactis *host.

### Functional characterization of the promoters PBM1, PB, and PS

**Figure 3:**
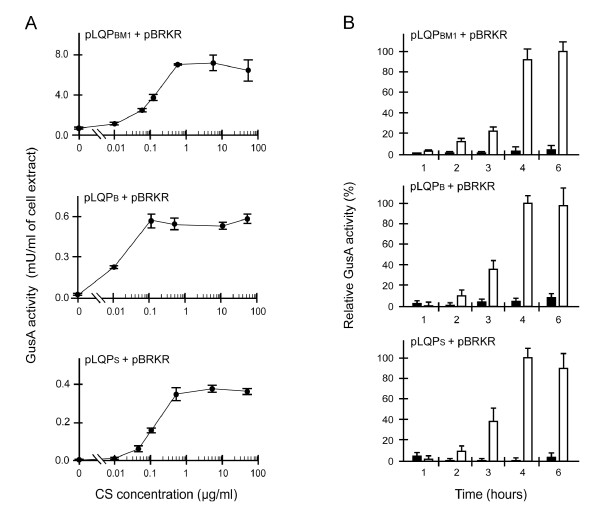
**Functional characterization of the CS-CbnK-CbnR system-controlled promoters PS, PB, and PBM1 in *Carnobacterium maltaromaticum***. Dose-response curves showing GusA activity as a function of CS concentration (A) and time course showing the GusA activity of cultures treated with 20 μg/ml of CS (white columns) or 0.05% trifluoroacetic acid (black columns) (B). In the time course experiment, the cultures were harvested for GusA activity determination at the indicated times. GusA activity was determined as described in Methods. Means ± standard error of triplicate assays are shown.

The promoters PBM1, PB, and PS have each two imperfect 10-bp direct repeats that are separated by a 12-bp AT-rich segment [18]. This sequence motif has been proposed to be a site for CbnR binding [18]. The sequence similarity of these promoters suggests that they have similar CS-dependent activation profiles. To investigate this possibility, the GusA reporter assay was utilized to characterize the CS-dependent activation patterns of these promoters in *C. maltaromaticum *LV17C transformants carrying pBRKR and either pLQPBM1, pLQPB, or pLQPS. We determined GusA activity in the cell suspensions of the transformants after treatment with CS at different concentrations (Figure 3A). The analysis revealed clear differences in the strength of the promoters PBM1, PB, and PS. In addition to this dose-response analysis, we investigated the time-dependent activation patterns of PBM1, PB, and PS by monitoring the GusA activity of cultures that were treated with CS at a single saturating concentration (Figure 3B). The kinetics of promoter activation was comparable for all the promoters, with GusA activity reaching maximum after 4 hours of CS treatment.

**Figure 4:**
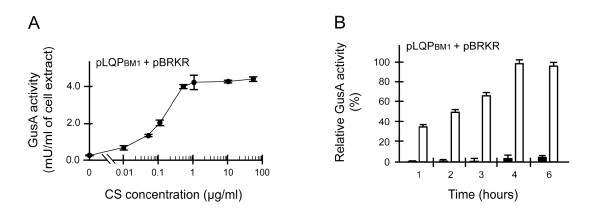
**Functional characterization of the CS-CbnK-CbnR system-controlled promoter PBM1 in *Lactococcus lactis***. Dose-response curve showing GusA activity as a function of CS concentration (A) and time course showing the GusA activity of cultures treated with 20 μg/ml of CS (white columns) or 0.05% trifluoroacetic acid (black columns) (B). In the time course experiment, the cultures were harvested for GusA activity determination at the indicated times. GusA activity was determined as described in Methods. Means ± standard error of triplicate assays are shown.

We also conducted dose-response and time-dependent experiments using *L. lactis *strains transformed with pBRKR and either pLQPBM1, pLQPB, or pLQPS. These experiments revealed a pattern of promoter activation in this heterologous host that is comparable to that observed in the homologous *C. maltaromaticum *host. Figure 4 shows the results obtained with the pBRKR/pLQPBM1 *L. lactis *transformants. Overall, our promoter functional analysis demonstrates that PBM1, PB, and PS displayed qualitatively equivalent regulation patterns, and that the functional properties of these promoters are not species specific.

## Conclusions

Deconvoluting the molecular bases of bacterial mechanisms of intra- and inter-species cell-to-cell communication is crucial to deepening our understanding of the social behavior of microorganisms, and to manipulate bacterial behavior in natural and artificial environments. In particular, a detailed knowledge of the molecular mechanisms of quorum sensing is anticipated to have practical relevance to medical, agricultural, and industrial applications. The present study was aimed at characterizing the molecular mechanism that is involved in quorum sensing-dependent regulation of carnobacteriocin production by *C. maltaromaticum *LV17B. This bacterium and its carnobacteriocins are of particular interest because of their potential application to food preservation. Our results provide evidence supporting a model in which the CbnK-CbnR two-component signal transduction system is necessary and sufficient to transduce the signal of the peptide autoinducer CS into activation of the promoters that drive the expression of the genes required for production of carnobacteriocins and the immunity proteins that protect *C. maltaromaticum *LV17B from their antimicrobial activity. The CS-CbnK-CbnR triad forms a three-component regulatory system by which production of carnobacteriocins is controlled in a population density-dependent (or cell proximity-dependent) manner. Notably, CS induces expression of its own gene, suggesting a positive-feedback loop that would result in a fast burst of gene up-regulation, which will lead to a sharp induction of carnobacteriocin and carnobacteriocin immunity protein production. The CS-CbnK-CbnR-based regulatory mechanism would permit the bacterial population to synchronize the production of carnobacteriocins and the carnobacteriocin immunity proteins. Such a population-wide action would afford a substantial burst of carnobacteriocin production that could increase the competitive fitness of the bacterium by enhancing its ability to inhibit the growth, or kill, susceptible bacterial competitors. Finally, the CS-CbnK-CbnR-based two-plasmid expression system we have constructed is a suitable genetic tool for undertaking future structure-function relationship analyses to map the residues required for the biological activity of CS, CbnK, and CbnR. This plasmid system also has potential for use as a starting point for the development of cloning vectors for inducible gene expression in *C. maltaromaticum, L. lactis*, and other lactic acid bacteria.

## Methods

### Bacteria, plasmids, and culture conditions

The parental strains and plasmids utilized in this study are listed in Table 1. *Escherichia coli *strains were cultured in Luria-Bertani medium (Difco) [25]. *C. maltaromaticum *and *L. lactis *strains were cultured in APT (Difco) and M17 medium supplemented with 0.5% (w/v) glucose (Difco), respectively, as described previously [15,24]. Ampicillin (Amp, 100 μg/ml), chloramphenicol (Cm, 10 μg/ml), and kanamycin (Km, 30 μg/ml) were added as needed to the culture media for selection and propagation of *E. coli *transformants. Chloramphenicol (5 μg/ml) and erythromycin (Em, 5 μg/ml) were added to the growth media for selection and propagation of *C. maltaromaticum *and *L. lactis *transformants as required.

### Genetic manipulation methods

General DNA manipulation techniques were performed using standard methodologies described previously [25]. Plasmid DNA was isolated from *E. coli, C. maltaromaticum*, and *L. lactis *strains by reported methods [17,24,25]. The isolation of genomic DNA from *C. maltaromaticum *was conducted as previously reported [15]. Transformation of *E. coli, C. maltaromaticum*, and *L. lactis*, as well as selection and screening of transformants, was performed by established procedures [17,24,25]. Restriction enzymes and other DNA-modifying enzymes were utilized as recommended by the manufacturers (Gibco, New England Biolabs). *Taq *PCR Core Kit (Qiagen) and Expand High Fidelity PCR System (Roche Applied Science) were used for polymerase chain reaction (PCR) as recommended by the manufacturers. The oligonucleotides utilized in this study were purchased from Integrated DNA Technologies and are listed in Table 2. PCR products were purified using QIAquick Gel Extraction Kit as recommended by the supplier (Qiagen). The nucleotide sequence of each of the PCR products cloned was confirmed by DNA sequencing at the DNA Sequencing Facility of Cornell University.

### Cloning of *Carnobacterium maltaromaticum *promoters

A ≈0.2 kb chromosomal fragment containing the predicted promoter upstream of* cbnBM1*, PBM1, was amplified using the *Taq *PCR Core Kit and the primers PcbnBMl-f and PcbnBMl-r. The PBM1 fragment was cleaved with *Bam*HI and *Eco*RI, and cloned into the screening vector pNZ273 cleaved with the same enzymes to insert the promoter upstream of the promoter-less *gusA *gene of the vector. The resulting plasmid, named pLQPBM1, was introduced into *E. coli*, then isolated from this host, and subsequently introduced into *C. maltaromaticum *and *L. lactis *by electroporation. A similar cloning strategy has been utilized to construct pLQPS and pLQPB, the pNZ273 derivatives carrying the promoters PS (located upstream of *cbnS*) and PB (located upstream of *cbnB2*), respectively, cloned upstream of the *gusA *reporter gene of the vector [19].

### Construction of plasmids expressing wild-type *cbnK *and *cbnR*

The plasmid pBRKR, expressing wild-type *cbnK *and *cbnR*, was constructed as follows. A 2.2-kb fragment encoding *cbnK *and *cbnR *was PCR-amplified from pLQB14 (vector pK194 carrying the 14-kb *Bam*HI fragment of pCP40) using the Expand High Fidelity PCR System and the primers BRcbnKR-f and BRcbnKR-r. The *cbnK-cbnR *PCR product was cleaved with *Eco*RI and *Hin*dIII, and then ligated into plasmid pUCl8 cleaved with the same enzymes. The resulting plasmid, named pCbnKR, was transformed into *E. coli*. Subsequently, the insert of pCbnKR was cloned into the expression vector pIL253 cleaved with *Eco*RI and *Hin*dIII to generate pBRKR. This plasmid was transformed into *L. lactis *and *C. maltaromaticum *strains.

### Construction of plasmids expressing mutated *cbnK *and *cbnR *variants

The plasmids pBRKA239R (with His 239 of CbnK replaced by Ala) and pBRKRN62 (with Asp 62 of CbnR replaced by Asn) were constructed as follows. The mutations in the regulatory genes were introduced in the pCbnKR backbone by fragment replacement using site-directed mutagenesis [26,27]. DNA fragments were amplified using the Expand High Fidelity PCR System. The CbnK Ala 239 mutant was generated with the primers BRBsmFI-f, BRH239A-f, BRH239A-r, and BRBsp-r. The CbnR Asn 62 mutant was created with the primers BRBsp-f and BRD62N-r. The resulting pCbnKR derivatives were transformed into *E. coli*. Subsequently, the mutated *cbnKR Eco*RI-*Hin*dIII inserts were cloned into pIL253 cleaved with *Eco*RI and *Hin*dIII to create pBRKA239R and pBRKRN62. These plasmids were transformed into *C. maltaromaticum *and *L. lactis *strains.

### Synthesis of the CS peptide

The peptide CS (SKNSQIGKSTSSISKCVFSFFKKC) was synthesized at the Peptide Synthesis Facility of the Rockefeller University (New York, USA) and purified to >97% homogeneity by standard reversed-phase high-performance liquid chromatography. The molecular weight of the purified peptide was verified by matrix-assisted laser desorption ionization time-of-flight mass spectrometry. Typically, purified CS was dissolved in 0.05% trifluoroacetic acid and stored at -80°C.

### β-glucuronidase assay

The GusA assays were performed as reported previously [19,24]. Briefly, the *C. maltaromaticum *and *L. lactis *strains were cultivated in APT and GM17 growth media, respectively, as described above. The cultures (2.5 ml, OD_600 nm _of 0.3) were treated with CS (from a stock in 0.05% trifluoroacetic acid) or 0.05% trifluoroacetic acid (negative controls). CS-treated and trifluoroacetic acid-treated cultures were incubated at 24°C *(C. maltaromaticum *strains) or 30°C *(L. lactis *strains) for 4 hours. After incubation, the cells were harvested, washed with buffer A (50 mM NaPO_4_, pH 7), and resuspended in buffer A to an OD_600 nm _of 2.0. The resuspended cells were permeabilized with acetone:toluene (9:1, 50 μl/ml of cell suspension). To assess GusA activity, 100 μl of the permeabilized suspension were mixed with 900 μl of assay buffer (buffer A with 10 mM β-ME, 1 mM EDTA, and 0.1% Triton X-100). The mixture was loaded (240 μl/well) in triplicate into 96-well plates containing 10 μl/well of a 100 mM stock of *p*-nitrophenyl-β-D-glucuronide (Sigma Chemical Company). The time-dependent change in absorbance at 405 nm at 37°C was measured in an ExpectraMax Plus Microplate Reader (Molecular Devices Corp.). The data were analyzed using SoftMax Pro software (Molecular Devices). Typically, initial rates (mOD_405 nm_/min) were calculated from ≥10 consecutive measurements over a 30-min period, and converted to GusA units using the excitation coefficient of *p*-nitrophenolate (18,500 M^-1^cm^-1^).

## Abbreviations

Amp, ampicillin; Cm, chloramphenicol; Em, erythromycin; GusA, β-glucuronidase; Km, kanamycin; OD, optical density.

## Authors' contributions

BHR carried out the strain constructions and conducted the strain phenotypic analyses. LENQ designed and supervised the study. BHR and LENQ wrote the manuscript. All authors have read and approved the final manuscript.

## Figures and Tables

**Table 1:** Bacterial strains and plasmids utilized in this study

**Bacterial strain or plasmid**	**Relevant characteristics**	**Reference, source**
**Strains**		
*Escherichia coli *DH5α	Cloning host	[25]
*Lactococcus lactis *MG1363	Gram-positive bacterium	[28]
*Carnobacterium maltaromaticum *LV17B	Carnobacteriocin producer carrying pCP40	[29]
*Carnobacterium maltaromaticum *LV17C	*C. maltaromaticum *LV17B derivative lacking pCP40	[29]
		
**Plasmids**		
pUC18	*E. coli *cloning vector, Amp^r^	[25]
pNZ273	*L. lactis *promoter screening vector, *gusA *reporter, Cm^R^	[24]
pIL253	*L. lactis *expression vector, cloned genes are transcribed from the *rep *promoter, Em^R^	[23]
pK194	*E. coli *cloning vector, Km^R^	[30]
pLQB14	pK194 carrying the 14-kbp *Bam*H1 fragment of pCP40	This study
pCbnKR	pUC18 with *cbnK-cbnR *insert, Amp^R^	This study
pBRKR	pIL253 expressing *cbnK-cbnR*, Em^R^	This study
pBRKA239R	pBRKR derivative with His 239 of CbnK replaced by Ala, Em^r^	This study
pBRKRN62	pBRKR derivative with Asp 62 of CbnR replaced by Asn, Em^r^	This study
pLQPBM1	pNZ273 with PBM1 promoter upstream of *gusA*, Cm^R^	This study
pLQPB	pNZ273 with PB promoter upstream of *gusA*, Cm^R^	[19]
pLQPS	pNZ273 with PS promoter upstream of *gusA*, Cm^R^	[19]

**Table 2:** Oligonucleotides utilized in this study

**Primer**	**Sequence**
BRcbnKR-f	5'-TAAAAGCTTTTGAATTCTGATATACTC-3'
BRcbnKR-r	5'-TCTCATCTTGCTGCAGGACAAATG-3'
BRH239A-f	5'-AGGAAATTTAAG**GCG**GACTATGAGAATC-3'
BRH239A-r	5'-GAGATTCTCATAGTC**CGC**CTTAAATTTCC-3'
BRD62N-r	5'-TGATTTAAATCGAT**GTT**TAAAAAGTAAATTC-3'
BRBsmFI-f	5'-GTTACTTTAGTATTGGGGACCG-3'
BRBsp-f	5'-GATACAAGGAACTTCTTCGAAAG-3'
BRBsp-r	5'-CTTTCGAAGAAGTTCCTTGTATC-3'
PcbnBM1-f	5'-CCGGGATCCTATGTCCTTTTGAAAAACCAAACTC-3'
PcbnBM1-r	5'-CCGGAATTCGTCTATACACTCCTTATAAAGTATTT3'

Restriction sites introduced with the oligonucleotides are underlined. Mutated codons are shown in bold.
